# The ABC of moral development: an attachment approach to moral judgment

**DOI:** 10.3389/fpsyg.2014.00006

**Published:** 2014-01-24

**Authors:** Aner Govrin

**Affiliations:** Department of Hermeneutics and Cultural Studies, Bar-Ilan UniversityRamat-Gan, Israel

**Keywords:** moral judgment, moral development, mentalization, infant development, social cognition, attachment theory

## Abstract

As with other cognitive faculties, the etiology of moral judgment and its connection to early development is complex. Because research is limited, the causative and contributory factors to the development of moral judgment in preverbal infants are unclear. However, evidence is emerging from studies within both infant research and moral psychology that may contribute to our understanding of the early development of moral judgments. Though its finding are preliminary, this proposed paradigm synthesizes these findings to generate an overarching, model of the process that appears to contribute to the development of moral judgment in the first year of life. I will propose that through early interactions with the caregiver, the child acquires an internal representation of a system of rules that determine how right/wrong judgments are to be construed, used, and understood. By breaking moral situations down into their defining features, the attachment model of moral judgment outlines a framework for a universal moral faculty based on a universal, innate, deep structure that appears uniformly in the structure of almost all moral judgments regardless of their content. The implications of the model for our understanding of innateness, universal morality, and the representations of moral situations are discussed.

Recent research in moral psychology has produced strong evidence to suggest that moral judgment is intuitive and is accomplished by a rapid, automatic, and unconscious psychological process (Damasio, 1994; Shweder and Haidt, 1994; Greene and Haidt, 2002; Hauser, 2006; Mikhail, 2011). This line of research challenged the long-dominant cognitive development paradigm conceived by Kohlberg (Piaget, 1965; Kohlberg, 1969; Turiel, 1983, 2006) according to which moral judgment is the product of conscious, effortful reasoning.

There is, however, considerable disagreement and confusion as to what moral intuitions are and how they work: what exactly are the underlying cognitive processes of these judgments that “operate quickly effortlessly and automatically, such that the outcome but not the process is accessible to consciousness?” (Haidt, 2001, p. 818). How are moral situations represented in our minds? What cognitive processes intuitively glue together different moral situations to one category?

In this paper, my main concern will focus on moral violations that involve harming others. Even though moral psychology and philosophy are broader than harm violations, it is likely that judgments about harm represent an important foundation of moral judgment (Nichols, 2004).

I will suggest that the patterns of people’s moral intuitions actually follow fairly straightforwardly from internally represented principles or rules acquired in infancy. My assumption is that moral judgment is a complex cognitive achievement that may rely on a set of building block systems that appear early on in human ontogeny and phylogeny. In this, I follow 20 years of infant research according to which the knowledge accumulated during the first year of life forms the foundation on which later learning, including language acquisition, counting, object categorization, social relations, and other complex cognitive skills rests (Starkey and Cooper, 1980; Wynn, 1990; Mandler and McDonough, 1998; Ensink and Mayes, 2010). According to this view, “in order to understand humans’ most complex cognitive skills, we should take a broad view and study not only adults who have mastered the skills and children who are acquiring them but also human infants and other animals. Although no young child or nonhuman animal possess these skills both exhibit many of the cognitive systems that serve as their building blocks” (Spelke, 2000, p. 1233).

The idea that our moral sense is essentially connected to early ties of dependency between the child and his or her caregiver is not new. It was proposed by John Bowlby’s attachment theory and Carol Gilligan’s ethics of care. Both theories emphasize the importance to moral development of the early relations between mother and infant (Bowlby, 1944, 1953, 1958, 1980; Gilligan, 1982; Gilligan and Wiggins, 1987). However, the ideas of attachment theory and ethics of care have not received the centrality in moral psychology appropriate to their importance.

Until the beginning of the twenty-first century, the central model in moral psychology was Kohlberg’s (1963a,b), according to which moral development is dependent on moral reasoning and on the growing cognitive faculties of the child at every stage of his maturation. Such a model is not relevant when it comes to the learning of moral principles in infancy prior to the development of a child’s faculties for abstraction and logical thought. Since the 1990s, moral psychology has radically changed its approach. On the basis of numerous works of research in the field, it was found that moral judgment is more a matter of emotion and affective intuition than deliberate reasoning (Greene and Haidt, 2002; Hauser, 2006). In the 1990s, the affective revolution was reinforced by a new focus on “automaticity”– the mind’s ability to solve many problems, including high-level social ones, unconsciously and automatically. This model suggests that moral judgments are best thought of as affect-laden intuitions. This is because they appear suddenly and effortlessly in consciousness, with an affective valence (good or bad), but without any feeling of having gone through steps of searching, weighing evidence, or inferring a conclusion (Greene and Haidt, 2002).

I suggest that advances in our understanding of the nature of moral judgment and the way affects organize the mind in the first year of life, offer us the opportunity to create closer ties between Bowlby’s theory of attachment, ethics of care, and moral psychology which were previously considered as belonging to separate domains.

The idea is to use evidence from research in moral psychology, infant research, and categorization, and then look at moral situations from a phenomenological perspective to show patterns and regularities. This integrated approach suggests that there is a universal, innate, deep structure that appears uniformly in the structure of almost all moral judgments indicating early origins. This deep structure bears the mark of the infant-caregiver dyad as the nucleus that is still present in the way adults represent moral situations.

## THE IMPORTANCE OF THE FIRST YEAR OF LIFE TO THE DEVELOPMENT OF KNOWLEDGE

Over the past decade, neuroscientific data has been collated from experiments involving both humans and animals (e.g., Teicher et al., 2002; Francis et al., 2003; Meaney and Szyf, 2005; Parker and Nelson, 2005). Though this data remains somewhat controversial (e.g., Wolff, 1996; Green, 2000), it appears to show the significant influence of early childhood experiences on the development of the brain and consequently its profound impact on social and emotional development.

Over the past 20 years, as infant research has become increasingly more sophisticated and complex, it has become clearer to researchers that infants possess a much more intricate and far richer knowledge of the world than had previously been assumed. These studies proved that:

(a) In the first year of life infants learn a great deal about the physical world, about the nature of objects, how they move and interact, and how they differ in their specific properties. (Gelman, 1990; Mandler, 1992; Spelke et al., 1992; Baillargeon, 1993; Bertenthal, 1993). Several lines of research suggest that at a very early stage in their development (3–6 months), infants may have ways of symbolizing basic kinds of social relations as well as an ability to perceive and analyze social events that are of significance to them. Infants aged between 7 and 12 months are able to understand that an action that is intentional is both rational and directed at achieving a specific objective.
(b) That knowledge accumulated during the first year of life forms the foundation on which later learning, including language acquisition, counting, object categorization, social relations, and other complex cognitive skills, rests (Starkey and Cooper, 1980; Wynn, 1990; Mandler and McDonough, 1998; Ensink and Mayes, 2010).

If that is so, then there is no reason for assuming that moral development differs from the progress of other key cognitive faculties. Given that dyadic interaction between infant and parent play a crucial role in the early organization of the mind (Beebe and Lachmann, 2002), there are grounds for assuming that this initial phase plays a significant role in the infant’s moral development, and that basic processes of dyadic interaction may shed light on how the infant learns the language of good and bad, right and wrong.

## THE IMPORTANCE OF THE FIRST YEAR TO THE UNDERSTANDING OF RELATIONS AND TO MORAL DEVELOPMENT

When does mentalization within the moral domain evolve in childhood, and what mental phenomena account for its appearance? Jean Piaget in The Moral Judgment of the Child (1932/1965), thought that mentalistic moral evaluation is a relatively late developmental achievement, emerging sometime around or after the age of 7. Piaget thought that children are initially “moral realists” who assign explicit blame based on objective states of the world, like outcomes, rather than on subjective states of the mind, like intentions. Yet, according to Hamlin (2013a), in the decades since The Moral Judgment of the Child (1932) a large body of work suggests that preverbal infants interpret agents’ object directed actions in terms of their goal-relevant traits, suggesting that at least some forms of mentalizing are present from early on in an infant’s development. By 3–5 months of age, infants pay more attention to events demonstrating mentalistic changes (changing one’s mind), than to physical changes (changing one’s motion); (see Woodward and Needham, 2009, for review).

In the moral domain, studies found that babies have an intrinsic moral foundation – the ability and readiness to judge what others do, a certain sense of justice, and an intuitive response to meanness. In several studies (Hamlin et al., 2007, 2010, 2011), infant participants between the ages of 6–10 months were shown a simulated three dimensional geometric “puppet show” in which geometric-shaped objects were used to role-play “helping/hindering situations.” The “helper,” a yellow square, assisted the circle up the hill; a red triangle (the “hinderer”), pushed the circle down the hill. Following the infants’ observation of the “geometric puppets,” the experimenter put the “helper” and “hinderer” on a tray and took them to the child. The researchers made a record of which of the two the child reached for, theorizing that what an infant of that age would reach for would be a reliable pointer to what he wants. The experiment revealed that 6- and 10-month-old infants consider an individual’s behavior toward others when assessing that individual as either attractive or, alternatively, aversive. In other words, infants showed a clear preference for an individual who was being helpful to someone else as opposed to one who placed obstacles in the path of another person. Moreover, the studies show that the participating infants preferred a helpful individual to one who stood on the sidelines and the latter to one who was obstructive.

Hamlin (2013a), also utilizing a puppet-choice methodology, provides evidence that 8-month-old infants incorporate, and even privilege, intentions in their social evaluations. In contrast, 5-month-old infants appear only able to distinguish characters who intend the outcomes they cause. Such results suggest that one requirement for mature moral judgments, the ability to distinguish between intentions and outcomes in morally relevant events, is present by 8 months of age. Also, infants prefer individuals who treat similar others well, and treat dissimilar others poorly (Hamlin et al., 2013). It was also found that 16-month-olds are sensitive to the pro- or antisocial behavior of a source that demonstrates preference for two novel foods.

When deciding what to eat, infants took into account the emotional reactions displayed by novel and previously prosocial sources, but not those of antisocial sources (Hamlin and Wynn, 2012).

The researchers note that these social evaluations share at least one crucial feature component with moral judgment: they do not stem from an infant’s own experiences with the actors involved. The infants themselves did not experience any consequences resulting from these “wrongdoings.” Their evaluations were made on the basis of witnessed interactions between unknown individuals. The infant, as an unrelated unaffected third party, is nonetheless making a judgment about the value of a social act.

These experiments show that infants have a certain type of knowledge that enables them to both distinguish between proper and improper behavior and understand social relationships on a basic level. Moreover, infants confronted with two novel agents with opposing goals are able, on the basis of their relative size, to anticipate the result of the first contest for dominance between them. Thus in their preverbal phase, it would seem that infants have the capacity to conjure up a mental representation of “dominance.” This representation uses an indicator that includes a phylogenic scale, which is metaphorically applied across all human cultures and languages to anticipate which of the two novel agents is likely to come out on top. (Thomsen et al., 2011).

However, the experiments tell us very little about the procedures or moral principles that infants learn, how they encode a moral situation, and what representations they compare it to.

Maternal care is the likely stimulus that enables an adult’s moral mechanism to develop. My assumption is that in precisely the same way as an infant born with innate linguistic faculties will only learn to speak if he grows up in the presence of people who speak to him, he will develop moral faculties only if in his experience someone else responds to his needs, takes care of him, and protects him at some level.

Even though researches were aware of the fact that moral and social faculties already begin to develop in the first year of life, they did not make room for, or place any importance on, the maternal role in this development or even consider it. In most of the studies, infants were still being perceived apart from their surroundings, as if they possessed an isolated mind that was developing separately from the environment in which they were growing up. For example, Hamlin et al. (2007, 2010), posit that the capacity of infants to evaluate individuals on the basis of their social interactions is unlearned (Hamlin, 2013b). Bloom (2013), claims that we have an in born moral sense that we spend the rest of our lives exercising in making judgments about good and evil. Likewise, Hoffman (2000) thinks that the empathic and social faculties of infants develop “naturally” without prior experience. Hamlin (2013a), thinks that though it is unlikely that young infants themselves benefit extensively from the basic ability to distinguish friends from foes, and arguably are too physically immature to profit from such an ability even if they had it, there are several examples of structures and capacities which emerge in development before they are specifically required.

So, today, even though an infant’s social and empathic abilities are a salient feature of any contemporary book on moral development, the role of maternal care in such development was not directly theorized upon. This lacuna has a long history in moral psychology that stretches from Piaget and Kohlberg to contemporary moral psychology. And yet, infant research conducted outside of moral psychology shows that out of all the influences around him, the one that affects the new born the most is the maternal care that he receives. Despite this consistent finding, theories in moral development failed to integrate it in meaningful ways.

## WHAT DO INFANTS LEARN FROM THEIR INTERACTIONS WITH THEIR MOTHERS?

There is a great deal of evidence that expectations of social relations emerge in the first months of life through infant-caregiver interactions. Beebe and Lachmann (2002), suggest that in the same way that infants categorize faces, shapes, objects, colors, and animals, they also form schemas or categories of interpersonal interactions (see Beebe and Stern, 1977; Stern, 1985; Beebe and Lachmann, 1988). A principle called ongoing regulations between mother and infant (Beebe and Lachmann, 2002) provides the most basic rule for organizing representations. The predictable ongoing regulations in mother–infant interactions create expectancies that organize the infant’s experience. The neonate detects “contingencies,” expected relationships between his own behavior and the environment’s reaction to it. An infant develops an ability to anticipate when something is likely to happen and an expectation that what he does has consequences. Thus, a remarkable set of pre-symbolic representational capacities exists in an infant’s first year (Stern, 1985; Beebe and Lachmann, 2002). The infant can sense whether or not the caregiver is acting contingently, and can determine whether behavior patterns are similar or different. The infant develops expectations of these patterns, remembers them, and categorizes them.

These anticipated outcomes are organized in terms of time, space, affect, and arousal. This is the equipment the baby uses to develop pre-symbolic representations of standard interactions well before language develops.

What is important here to emphasize is that in the early stages of life, infants do not learn at the level of content. They learn procedures, patterns of interaction, and a shared system of rules for maintaining the management of joint actions in the first year of life. (See for example: Bakeman and Brown, 1977; Bruner, 1977, 1983; Stern, 1985, 2002; Cohn and Tronick, 1989; Tronick, 1989).

## THE ATTACHMENT APPROACH TO MORAL JUDGMENT: A PHENOMENOLOGICAL PSYCHOLOGICAL ANALYSIS OF MORAL SITUATIONS

In order to establish the link between early infancy and the acquisition of basic moral faculties, we must be capable of defining: (1) the appropriate stimulus that is likely to lead to the learning of the proper processes by which moral judgment is exercised and, (2) the deep structures that are common to the entire range of moral situations, including the link between those structures and the initial stimulus that made moral learning possible.

How can these assertions be tested?

There is no direct evidence of the way in which infants learn moral principles, just as there is no direct evidence of the way in which infants learn the deep structures of language. Therefore, we must discover the deep structures of moral situations and then look into the way in which these are linked to the first year of life.

The goal is to posit the most minimal set of assumptions that can still account for various moral judgments and situations.

## THE DYAD SUPERIORITY EFFECT OF MORAL SITUATIONS: GRAY’S FINDINGS

The features of moral situations to be discussed are simple and obvious. It will quickly become apparent that, paradoxically, due to their basic, simple and intuitive nature, these features go mostly unnoticed. As a result, we hardly discern them or give them much thought.

We will want to understand what characteristics different moral situations have in common. How do people recognize moral situations and notice regularities within them? What are these regularities? How are moral situations represented in our minds? What kind of categorization do we use when processing a moral judgment?

What then is the most invisible and yet the most salient characteristic of a moral situation?

*The fundamental unit of moral situations is the dyad.* I term this phenomenon the *dyad-superiority effect* of moral situations. Essentially this means that moral situations are mentally represented as two parties in conflict.

We have strong support for the dyadic nature of moral situations. A series of studies by Gray et al. (2012), showed that moral judgments do not depend merely on the superficial properties of moral events but also on how those events are mentally represented. Gray conducted a large-scale survey which investigated specific links between mind perception and morality. Respondents evaluated both the mental capacities of diverse targets (e.g., adult humans, babies, animals, God) and their moral standing (Gray et al., 2007). In particular, participants assessed whether target entities deserved moral rights and whether they possessed moral responsibility. The mind survey revealed that people perceive minds along two independent dimensions. The first dimension, *experience*, is the perceived capacity for sensation and feelings (e.g., hunger, fear, pain, pleasure, and consciousness). The second, *agency*, is the perceived capacity to intend and to act (e.g., self-control, judgment, communication, thought, and memory). An entity can be high on both dimensions (e.g., adult humans), low on experience and high on agency (e.g., God, Google), high on experience and low on agency (e.g., children, animals), or low on both (e.g., the deceased, inanimate objects). The mind survey demonstrates key connections between mind perception and morality.

Gray found that the essence of moral judgment is the perception of two complementary minds – a dyad of an intentional moral agent and a suffering moral patient. One of Gray’s most important findings is that moral judgment is rooted in a cognitive template of two perceived minds – a moral dyad of an intentional agent and a suffering moral patient (Gray and Wegner, 2009). According to Gray, the essence of morality is the perceived interaction between minds.

Agency qualifies entities as moral agents – capable of doing good or evil – whereas experience qualifies entities as moral patients – capable of benefiting from good or suffering from evil. Adult humans usually possess both agency and patiency, and can therefore be both blamed for evil and suffer from it. A puppy, according to Gray, is a mere moral patient; we seek to protect him from harm but do not blame him for injustice.

Gray posits that despite the variety of moral transgressions, the moral dyad not only integrates across various moral transgressions but also serves as a working model for understanding the moral world. This dyadic template fits the majority of moral situations because mind perception is as flexible as moral judgment itself. A dyadic template of morality suggests that people are categorized as either moral agents or moral patients – a phenomenon called moral typecasting. Moral typecasting also influences our perception of the target person’s mind. When someone is categorized as a moral agent, observers automatically infer the capacity for agency. This means that simply doing something good or evil can bring with it corresponding attributions of intention, especially evil intentions (Knobe, 2003; see Gray and Wegner, 2009). Likewise, when someone is categorized as a moral patient, people automatically infer the capacity for experience and greater sensitivity to pain (Gray and Wegner, 2009).

Gray posits that the essence of morality is expressed by the combination of harmful intent and painful experience. If so, acts committed by agents with greater intent and that result in more suffering should be judged as more immoral.

Following Gary, I suggest that a dyadic structure is the most common trait of moral situations. A dyad is present in the background of every moral situation regardless of whether it involves many parties, or a group (several individuals, large groups, or even nation states).

Second, the bedrock of most moral judgments is an observer examining a dyad. The word “observer” is, in a sense, misleading because people make moral judgments both as observers and as participants. I use the word “observer” only for demonstrative purposes. At this stage, I am interested in understanding what leads us to judge theft or medical negligence as a wrongful act, rather than the way in which the thief or his victim judge the situation. This also resembles the experiments of infants’ moral judgment (Hamlin et al., 2007, 2010) in which infants were observers. Thus, within a basic moral judgment situation three sides are involved: two conflicting parties (a dyad) and an observer.

**O relates to the following dyad: A → C**

**O – Observer**

**A Perceived wrongdoer**

**C Perceived victim**

**→ Behavior, Harm done, Overall attitude of A to C**

In the following examples I demonstrate how moral situations remain constant in their dyadic structure across a wide range of moral dilemmas of entirely different content and associative nets. The question mark signifies that the moral judgment is in question.

(a) Murder/Manslaughter case
Did John kill David in cold blood or did David provoke him prior to the killing?
Observer (O) relates to the following dyad:
(A) David → (?) (C) John.
(b) Bombing civilians in self-defense
Does a state have the right to bomb civilian neighborhoods in a neighboring state from which militants have fired rockets into its territory killing civilians?
Observer (O) relates to the following dyad:
(A) State’s army → (?) (C) Civilians of neighboring state.
(c) Medical negligence case
Were the medical complications suffered by the patient after surgery caused by the physician’s negligence?
Observer (O) relates to the following dyad:
(A) Physician → (?) (C) Sick patient

Note that the questions relate to different issues. Some, like those relating to medical negligence cases are questions about facts. Others are questions about personal beliefs and values. The bombing of civilians in populated areas from which missiles were fired raises especially important issues. As is the case with many other moral dilemmas, the observer has to choose between two conflicting sides which I have chosen to represent in this instance by the letters A and C. Many serious moral dilemmas arise from a conflict between two or more deeply felt obligations pulling in opposite directions toward two parties each of which is perceived as having been harmed.

In fact, in such situations two dyads are presented to the mind:

(A) State’s army → (?) (C) Civilians of neighboring state.

And

Militias → (?) (C) Civilians of state (A)

Thus, the process of reaching a judgment involves deciding which party you side with. In order for a judgment to be made, one of the dyads has to succeed in capturing the observer’s mind while the other is discarded.

This example shows that the complex social reality provides us with moral dilemmas that are manifestly more complex than a simple dyadic component.

However, dealing with these dilemmas can only be accomplished by breaking down their complexity into simple sub dyads.

I suggest that the process of construing a dyad when presented with social information about conflict, probably occurs at a very early stage in the processing of information; that several pieces of information relating to each party can be evaluated simultaneously; and that the basic process is fast, unintentional, efficient and occurs outside awareness.

## DECODING MORAL SITUATIONS

Thus far we have seen that breaking down a moral situation into a basic dyad enables us to deal with a vast amount of complex material relatively quickly.

Our judgment of different dyads seems to be fairly flexible and encompasses an astonishing range of situations. In fact, one of the most striking facts about our human morality is that people can morally judge an unlimited number of dyads on an unlimited numbers of topics.

In this section I will attempt to unravel the procedures whereby the moral judgment is reached.

Given the enormous amount of data that exists in relation to any given moral dyad, how do we organize the information for a particular perceived dyad? How do we extract a judgment from the basic features of A, C, and →?

It is probably the case that in the process of forming a moral judgment the moral dyad that appears in our minds is evaluated against some prior knowledge we possess about dyads. My assumption is that we can only make a moral judgment if, in our minds, we hold some reliable form of *prior knowledge representation* of the moral situation, a mental form for what we know about conflicts in our social environment.

Thus, I assume that we deal with moral situations in the same way we deal with other concepts. We categorize the situation as moral and then judge it according to the pre-existing representation it most closely resembles (Hahn and Ramscar, 2001).

Before describing the primary component of that dyad, it is important to understand what criteria are most often applied in judging moral situations. Intentionality and controllability seem to be crucial for moral judgments. There is a consensus among professional and lay evaluators of human behavior that to praise or blame an agent, the agent must have acted intentionally, with foresight of the consequences, and must have caused the outcome (Shaver, 1985; Schlenker et al., 1994; Alicke, 2000; Weiner, 2006; Alicke and Rose, 2010). Full responsibility inferences require internal and controllable causality, intent, and the absence of mitigating circumstances.

My contention is that the various components of a moral situation such as intentionality, controllability, personal responsibility and free will, have an additional layer of representational content that hasn’t been noticed by social psychologists. They are secondary features. They represent something more primary, more basic.

The key underlying thesis that I will present is that the most informative features of moral judgments – intent, free will and controllability – are underpinned by a more profound feature – our knowledge about infants (or children) and adults.

We have an affective and cognitive mechanism that is highly sensitive to the distinctions between child-like and adult-like traits. As we will see, these traits are highly informative for our understanding of others.

The same parameters that are crucial to the attribution of responsibility for a wrongdoing (intentionality, controllability and free will) are those that are crucial to the distinction between children and adults.

I suggest that we represent each of the parties (A, C) in ways that are comparable to our representation of children and adults. All our efforts are geared to construct the reality of the moral situation in terms of an adult–child dyad. Judgments placing the parties on the child–adult spectrum, come to mind quickly and effortlessly, seemingly popping out of nowhere, without much conscious awareness of their origins or of the manner of their formation.

## REPRESENTATIONS OF CHILD-LIKE AND ADULT-LIKE TRAITS

To simplify things, I will use here the theory of schematic representation (Rumelhart and Ortnoy, 1977) as a simple model for understanding moral judgments. I have chosen schemas because they are often more task-oriented than exemplars or prototypes and are less concerned with recognition and classification. Rather, a schema is a mental framework for organizing important knowledge, creating a meaningful structure of related concepts based on prior experiences. Therefore, schemas seem more appropriate to the moral domain which involves not merely recognition and classification but also organizing material in a particular way.

Schemas have received significant empirical support from studies in psycholinguistics and cognitive psychology (Bartlett, 1932, 1958; Rumelhart and Ortnoy, 1977; Mandler, 1984; Komatsu, 1992).

Construing the two conflicting parties as a child–adult dyad probably activates a particular schema that is common to most people, and sufficiently wide ranging to be applicable to a broad variety of specific moral situations.

## HOW DO WE JUDGE A CONFLICTED DYAD?

The basic idea is that non-conscious judgments of a dyad are formed automatically, effortlessly, ubiquitously and rapidly, before any conscious processing has taken place. In generating a non-conscious moral judgment, we perform two mental operations:

(a) Evaluating the child-like and the adult-like characteristics of each party and deciding, if we are able to, which of the parties matches an adult schema and which a child schema. As we will see, the most salient feature that differentiates between children and adults is dependency.
(b) Evaluating the relationship between the adult and child-like parties in terms of (→) where → is the symbol for the harm done and the overall relation of the independent *vis-a- vis* the dependent in a particular dyad. That is, we do not have schemas only for children and adults.

We also possess a schema for the dyadic relation, centered on our knowledge of adult obligations to children. So the moral judgment is much more than an evaluation of the harm done. It is the evaluation of the overall attitude of A to C based on our prior expectations of how adults should treat children.

## THE EVALUATION OF THE CHILD-LIKE AND ADULT-LIKE CHARACTERISTICS OF EACH PARTY

The evaluation of the moral situation is a derivative of our inner schemas of children (dependents) and adults (independents). In our minds, different expectations, feelings, cognitions, and mental images, are associated with children and adults.

For example, we are emotionally much more responsive to the suffering of children than to that of adults.

Dijker (2001), showed wounded bodies of people of different ages all supposedly involved in a car accident. It was found that toddlers are seen as more vulnerable than pre-adolescents who, in turn, are viewed as more vulnerable than adults. Elderly persons are perceived as more vulnerable than adults but not as vulnerable as toddlers.

The fact that the reactivity to old people’s suffering is also high shows that age, as an isolated characteristic, is not the crucial issue. Rather it is the victim’s child-like/dependent/vulnerable/weak quality- in this instance among elderly people.

Strong emotional reactivity to children’s and infants’ suffering is not the only thing that distinguishes the way in which we relate to children and adults. Apparently children and adults are identified by separate schemas that also involve cognitions and attributions. Children are perceived as weak, needy, helpless, lacking control, vulnerable, dependent on others, and unable to take care of others. These traits have profound implications on our attributions and moral judgments.

Given our schemas for children, in our cognitive system infants/children (dependents):

Cannot discriminate between right and wrong.

Are less responsible for their actions even when those actions are harmful.

Are not fully aware of the consequences of their behavior.

Do not intend to harm (in the way adults do).

Do not control the happenings in which they are involved.

Cannot take care of someone else who is in need.

Are helpless to the extent that their basic needs have to be supplied by a caregiver in order for them to survive.

Usually evoke positive feelings of tenderness, caring, and empathy, when they are distressed.

Thus, for example, when an infant scratches his mother’s face or lets a full glass of water fall off the table, we don’t consider him as having consciously intended to cause the damage.

According to our schemas for adults (independents), the very opposite set of propositions is true. We think that unlike children, adults have personal control of their behavior, do discriminate between right and wrong, and are responsible for their actions.

In the process of moral judgment the more someone matches a child-like schema (C), the *less likely* it is that the observer will:

Have a high assessment of intentionality, controllability, foresight, free will and causation.

Hold him responsible for his actions.

Tend to think that he has acted of his own free will.

Respond to him negatively.

And *the more likely it is* that the observer will:

Understand his behavior and forgive his wrongdoing.

Be more sensitive to his suffering.

Alternatively, the more someone in a moral situation matches an adult-like schema (A) *the more likely it is that* the observer will:

Have a high assessment of intentionality, controllability, foresight, free will and causation.

Hold him responsible for his actions.

Consider him to have acted with intent, deliberation, premeditation and malice.

Not forgive or understand A’s wrongdoing.

Be less sympathetic to A’s suffering.

The two schemas are different points of location on one continuum that can encompass a large variety of situations. The schemas are fixed around defining features of adults and children such as big/small, weak/strong, vulnerable/ resistant, helpless/powerful, dependent /independent, knowingly/unknowingly, responsible/irresponsible. The schemas are broad enough to handle endless variations of these themes. I suggest that when facing a moral situation, the mind uses these schemas to select and organize the information that will most effectively aid us in the judgment process.

As I stated above, it is important to note that the schemas we use in evaluating each party are not related to how children and adults *really are.* Parents, teachers, and anyone who has early memories of what it is like to be a child, realize that children can at times be cruel, aggressive and hostile toward other children and adults. Numerous studies have demonstrated that from an early age the aggressive behavior of children is expressed in all sorts of different ways (Crick, 1996; Crick et al., 1997).

Adult-like or child-like dimensions are not necessarily related to specific age but to the quality of a person or interaction. To put it more accurately, we are looking for cues of dependency and independency. For example, people unconsciously associate disability with child-like features (Robey et al., 2006). For example, college students spoke to others who they believed to be adults with disabilities much as they did to the 12-year-old child (Liesener and Mills, 1999).

In attributing child-like features, age is not important *per se* but as a sign of dependency/ independency. Parents who become angry with a 3-year-old child who is pulling at his baby sister’s hair, and who threaten to punish him, believe that the child is sufficiently independent to be capable of restraining himself, show sensitivity toward his sister, and stop harassing her. They regard him as responsible and able to exercise self-control and free will. Under these circumstances a 3-year-old is recognized as A.

**FIGURE 1:**
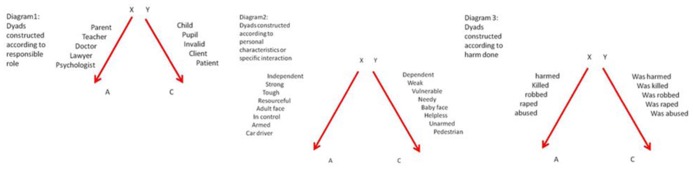
**Constructing dyads.** The sudden appearance in consciousness of a moral judgment first involves construing two asymmetric parties as child-like (dependent) and adult-like (independent). We construct these categories according to particular cues such as the responsible role of one party toward the other (diagram 1), personal characteristics of each party, or according to a particular interaction (diagram 2), or the harmful act itself (diagram 3).

As shown in **Figure 1**, there are at least three possible ways by which we decide who the dependent is and who the independent: role (diagram 1), personal characteristics (diagram 2), and harmful act (diagram 3).

The “detection” of child-like and adult-like characteristics is not entirely rational and not always relevant. For example, a number of experiments (Berry and Zebrowitz-McArthur, 1985) indicate that baby-faced people are less likely to lose their case than people considered to have a mature face (Berry and Zebrowitz-McArthur, 1988; Zebrowitz and McDonald, 1991). However, this influence also appears to depend on the nature of the offense. A baby-faced defendant will be considered less likely to have committed an offense intentionally, and more likely to have committed an offense by negligence than a defendant with a mature face.

The evaluation of child-like and adult-like characteristics in a particular moral situation is observer relative. The same person in a particular dyad might be construed as A by one person and as C by another. In fact, *construing the parties as C or A is the dominant act of moral judgment.* If party X matches an adult schema (A), and party Y matches a child schema (C), it means that we think X has done harm to Y and that we sympathize with C and condemn A. But this is only true for an observer that perceives X as adult-like and Y as child-like. So, child-like and adult-like schemas are not just cognitive assessments of traits. They incorporate our emotions, judgments, and actions toward the parties.

Whilst the decision as to which party is C or A is highly subjective, *the general traits within us that are associated with children and those associated with adults are constant and universal*. That is to say, our schemas for dependents and independents are the basic building blocks of a universal morality. These schemas are used differently by different cultures and peoples and yet one cannot construct a moral judgment without them.

## EVALUATING THE RELATIONSHIP BETWEEN THE ADULT-LIKE AND CHILD-LIKE PARTY (→)

Even if we match each party to adult and child schemas, the judgment remains incomplete. We do not simply compare the two parties individually and decide which one is more helpless, needier, or more powerful. Our judgment depends on something much more profound. It is linked to the *nature* *of the dyadic relations*. Just as we have different schemas for adults and children, so we have a schema for the dyadic relations between them.

This representation consists of our expectations of what adults should and should not do to children. Adults have obligations toward children and we seem to know these obligations intuitively.

**FIGURE 2:**
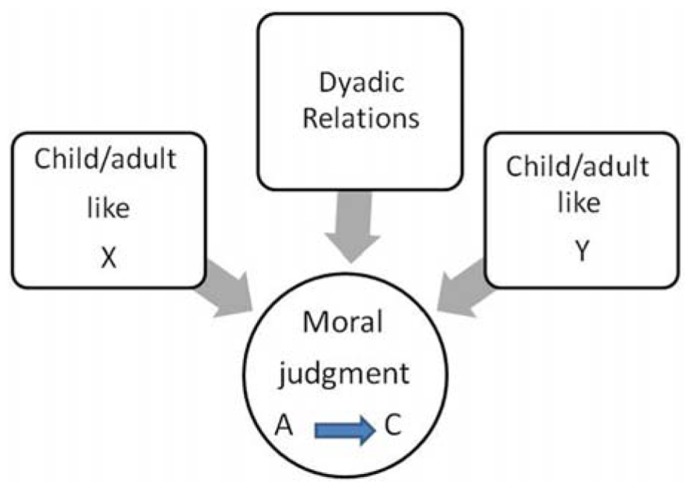
**The attachment model of moral judgment.** In generating a non-conscious moral judgment, we perform two mental operations: we impose a dyadic structure of child–adult/agent–patient (Gray et al., 2012) on two parties in conflict and we compare the behavior of A toward C with our prior expectations of what adults should and should not do to children. Acts that violated our expectations are judged as morally wrong. Whilst the decision as to which party is C or A is highly subjective, the general traits that are associated with children and those associated with adults are constant and universal.

The question that needs to be asked is this: how did the perceived adult-like party relate to the child-like party during their interaction? This criterion only concerns the perceived adult-like party (A) since we infer from our schema for the child–adult dyad that children are not expected to take care of anyone. That is why the moral situation is construed as A → C and not A → C or C → A. The one-way direction signifies the asymmetry between C and A. Thus, in the course of evaluating each party’s characteristics as child-like and adult-like, much weight is given to the evaluation of A’s actions and his awareness of the dependency of the other party (see **Figure 2**).

Let us turn again to another one of our earlier examples – medical negligence. Was the physician’s negligence responsible for the medical complications suffered by the patient after surgery?

A quick and effortless analysis reveals that the physician matches the adult schema and the patient the child schema because the sick patient depends on the physician and not the other way around. The dyad therefore consists of a physician mentally construed as A, and a patient mentally construed as C.

However, the judgment process is not complete without the evaluation of →. In the next evaluative stage our prior expectations of A in the presence of C become activated and interact with what we know about doctors and their obligations toward patients. Only if the physician’s actions failed to meet our expectations of adults in the presence of children will we judge the case to be one of negligence.

We compare a doctor’s actions with our expectations of him: he should examine the patient thoroughly; he shouldn’t opt for medical procedures that will endanger the patient’s life; he shouldn’t leave the operation in the middle of a procedure because he has to get to a show in the evening. Our entire moral judgment might change if it transpired that the doctor’s negligence resulted from him having had a heart attack during surgery. In such circumstances he may be perceived as highly dependent and his negligence may be viewed less severely.

## CONSTRAINTS

**FIGURE 3:**
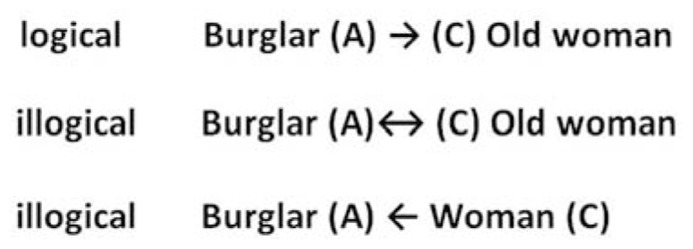
**The mental representation of A → C CONSTRAINTS our cognitions and emotions.** Once people construe the parties as A → C, the pattern of moral judgment follows a specific direction to the exclusion of all others.

In the process of forming moral judgments, especially in severe harm norm violations, the observer will have contrasting cognitions and emotions toward each of the parties to the conflict (see **Figure 3**). A mental representation of A → C clearly directs our cognitions and emotions. A constraint is a kind of rule that places extra conditions on dyadic structures. When the moral judgment is unambiguous and the harm is judged as serious, the observer will experience negative emotions such as blame and rage toward A, and positive feelings such as compassion, empathy, and pity toward C. The affective response matches a set of cognitive convictions related to the question of which party is wrong, needs help, deserves punishment etc. Observers might react with different levels of emotional intensity because individuals differ in their sensitivity to these vital cues of wrongdoing. However, both affect and cognition will follow one fixed, particular, direction. Construing the two parties as A → C imposes constraints that moral judgment must necessarily satisfy.

Suppose the mind is presented with the following information: “a man stole money from a poor, elderly woman.” The observer construes the situation as:

**Man**→**elderly woman Stealing**

The implication of construing the moral situation in this manner is that the observer’s affective system responds by feeling sorry and showing concern for the elderly woman and/or by condemning the burglar. Some observers might respond with sorrow or extreme rage; others will be completely indifferent, and most will react moderately. Of course, numerous personal, social, contextual, and cultural elements determine the observer’s response and its intensity. For the moment, however, I want to concentrate on the fact that though people differ in the intensity of their affective response, the *direction* of both the affective and the cognitive reaction is similar if, and only if, the observer construed the situation as A → C.^1^

Once people construe the burglar and the old woman as A → C, the pattern of moral judgment becomes fixed and constant. People will not condemn the elderly woman and empathize with the burglar. Therefore, the negative and positive feelings, as well as the feelings of those who remain relatively indifferent, follow a specific direction to the exclusion of all others.

Contrary to what the accounts of sentimentalists or intuitionists may argue (Prinz, 2007; Haidt, 2010) our moral judgments, unlike our esthetic tastes, are not arbitrary. Even when they lead to contradictory conclusions, they are not entirely flexible. Though it can be said that the construing of moral judgment is tolerant and allows for diverse cultural and personal projections, it is not the case that each and every projection will be perceived as sensible and/or acceptable. *A dyad will allow certain projections and block others if, and only if, it is construed as A → C*. Moral judgments are therefore constrained by rules that are guided by the knowledge we have as to how dyads should function and work. Our expectations of perceived independents in the presence of dependents impose extrinsic requirements on our moral judgments.

The greater the harm norm violation, the more the parties become polarized on the child-like and adult-like continuum. The more the perceived adult-like party has failed to fulfill an obligation toward the child-like party, the more the judgment of him will tend to be negative. However, the more either one of the parties, or both, are viewed as possessing a mixture of child and adult-like characteristics, the more ambiguous will be the judgment.

The general idea is that moral judgment involves computing child-like and adult-like characteristics. This process is almost reflex-like: it is fast in operation and automated so that, for instance, one cannot help taking into account the child-like face of an adult, the young age of a thief, or the unintended harm of an accidental killing.

## THE NEURAL WORKING OF MORAL JUDGMENTS: TWO TENTATIVE SUGGESTIONS

### MORAL JUDGMENT AND THE HUMAN VISUAL SYSTEM

If humans treated every new right-wrong situation as a new and unique experience, we would quickly drown in a baffling and confusing state of mind. The attachment approach to moral judgment suggests that to improve the situation, the cognitive system groups moral situations into the meaningful category of the A → C format. The various parts of the moral situations are perceived holistically rather than separately or independently. The different features of moral situations such as intention, harm, asymmetry of force, child-like/ adult-like characteristics of each party, are incorporated within the whole format of A → C. If David used a pistol to kill John in cold blood then the taking of life, the intentional act and the asymmetry of force, lead the different elements of this dyad to be grouped into a severe moral violation. This judgment has properties (such as strong condemnation of David) not possessed by any individual element.

Gray et al. (2012), suggest that if our template of morality is dyadic – perceived intentional moral agent and a suffering moral patient – we should be compelled to complete the moral dyad when it appears incomplete. For example, when we see someone blameworthy – an apparent moral agent – we should complete the dyad by inferring the presence of another mind to which is suffering – a moral patient. Gray suggests the phenomenon of dyadic completion occurs at an intuitive level – like the Gestalt completion. Here are some examples of dyadic completion Gray cites. In one study, participants received electric shocks that were administered either intentionally or accidentally, and though the shocks were identical in voltage, the more intentional (and blameworthy) shocks were experienced as physically more painful (Gray and Wegner, 2008). This increased experience of pain from intentional shocks also translates into increased skin conductance responses (Gray et al., 2012). Intentions are so strongly linked to culpability that even irrelevant intentions can increase judgments of culpability. For example, people forced to kill others at gunpoint are perceived as more morally wrong when they wanted the man dead, even though they had no choice (Woolfolk et al., 2006). Unrelated bad intentions can also make an act blameworthy. Alicke (1992) found that people attribute more culpability for ignoring a stop sign when the driver is hurrying home to hide drugs rather than to hide an anniversary present for his wife.

It is unknown what neurobiological framework can account for the dyadic completion. Most cognitive psychological moral theories are formal and detached from neuroscience. I suggest that much can be gained by taking advantage of the large amount of information available on the neurophysiology of visual recognition. Although moral judgments and visual recognition are separate unrelated domains, what might be of interest to us is the ability of the brain to complete missing elements so that recognition remains largely unaffected by such obstacles. Basically, the thinking is that visual images constructed by the brain are holistic- i.e., are far above what is expected from the linear sum of individual components. Human brain imaging research has strongly supported such holistic aspects by showing that one cannot explain the neuronal activity measured in high order visual areas in response to a picture as a sum of the responses to the picture elements.

Although visual recognition is a perceptual phenomenon, it can also be viewed as an ubiquitous property of various types of neural network models (Williams and Jacobs, 1997; Ullman, 1998). Such networks, upon presentation of a partial input pattern, can settle quite rapidly into an attractor state corresponding to the complete stored pattern (Lerner et al., 2002).

Studies point to the lateral occipital complex (LOC), as a central site in which object completion effects are manifested. In one study (Lerner et al., 2002), subjects were presented with three types of images: (i) whole line drawings of animal or unfamiliar shapes (“whole”); (ii) the same shapes, occluded by parallel stripes which occupied roughly half of the surface area of the images (“grid”); and (iii) the same stripes, “scrambled” so that the relative position of the regions between the stripes was changed while the local feature structure remained intact (scrambled). Behavioral measurements showed a high degree of object completion in the “grid” condition, but not in the “scrambled” condition. The functional magnetic resonance imaging (MRI) results show a significantly higher activation of the “grid” images compared to the “scrambled” images in the LOC. Other studies show that infants only a few months old complete representations of objects behind occluders (Kellman and Spelke, 1983), and psychophysical experiments on adults suggest that such completed representations determine the allocation of visual attention (He and Nakayama, 1992).

Taken together, these results make important progress in situating the LOC on a continuum of possible shape computations: after contour completion, border ownership, and invariance to form cues, size and position are attained (Kourtzi and Kanwisher, 2001). It is plausible to think that in the same way that areas in the brain play a critical role in object completion, other areas are dominant in the completion of the dyadic Gestalt.

But moral judgment is much more dynamic and complicated than object recognition and encompasses a huge amount of computations of various kinds. How does the neural code for moral situations take into account so many considerations in such a short time to form an holistic impression? How does the holistic judgment emerge?

### CONNECTIONISM

According to the proposed model, moral judgments are accomplished by a dynamical system in which they gradually emerge through ongoing cycles of interaction between evaluations of the two parties and their relations in terms of (dependency/independency, weak/strong/ like-me, not like-me, intentional/unintentional, mild harm/severe harm etc.). Thus, multiple sources of information – both bottom-up cues and top-down factors – powerfully interact and integrate over time to produce moral judgments.

The end-result of these evaluations is to determine who is A, who is C, and what kind of violation happened between the two. As such, this system permits lower-level sensory perception and higher-order social cognition to continuously coordinate across multiple interactive levels of processing to give rise to stable moral judgments.

What model of the brain can describe such a hypothesis? Some researchers (Hopfield, 1982; Rumelhart et al., 1986; Thagard, 1989, 2000; Harman et al., 2010; Freeman and Ambady, 2011) have argued that a connectionist network model possibly provides us with a way of explaining how people reach judgments about others. Dynamical systems, such as a recurrent connectionist network of the human brain, are powerful in their ability to integrate multiple simultaneous sources of information. In a recurrent connectionist network, there are a number of nodes with connections that can be positive (excitatory) or negative (inhibitory). Positive links connect one set of nodes to other nodes so that as one of the nodes becomes more excited, its excitation increases the excitation of the other nodes (Freeman and Ambady, 2011). Conversely, as the excitation of one such node lessens, or is in receipt of negative levels of excitation, the excitation of the other nodes is lessened. Negative links connect nodes so that as one node receives more excitation, the others receive less, and *vice versa*. When applied to elements in a moral situation positive and negative excitation cycles will circulate in the network and a steady state will be achieved resulting in a full gestalt of the moral situation as A → C.

Because a node’s activation is a function of all the positive and negative connections to other nodes that are activated in parallel, the final activation of a node (i.e., at the point at which the system stabilizes) can be thought of as the satisfaction of multiple constraints. In a connectionist model, each connection between nodes is a constraint (Freeman and Ambady, 2011). For instance, a node representing the category baby face might excite and be excited by another node representing the cognition “wrongdoing was unintentional.” When these two nodes are incorporated in a larger recurrent network that is stimulated by, for instance, a baby face, this baby face – unintentional connection between nodes serves as a constraint on the network. That is, for the network to ever achieve stability, activation must flow through that connection and incorporate it into an overall stable pattern that includes all other nodal connections.

Thus, nodes in a recurrent network constrain each other in finding the best overall pattern that is consistent with the input. Such a model of a connectionist network can explain how one single component of a dyad influences the other components. For example, many studies have proved that in cases in which the severe harm was entirely accidental, people are nevertheless willing to say that the agent is culpable and that the severe harm caused is his responsibility (for a review and meta-analysis, see Robbennolt, 2000). We can understand this by assuming that a certain component of the dyad, in this case severe harm and extreme suffering, strongly activates the other nodes and skews them in a certain direction. Again we are confronted by the role of dyadic Gestalt: when harm done is moderate that node is less active so that attribution of responsibility lessens.

**FIGURE 4:**
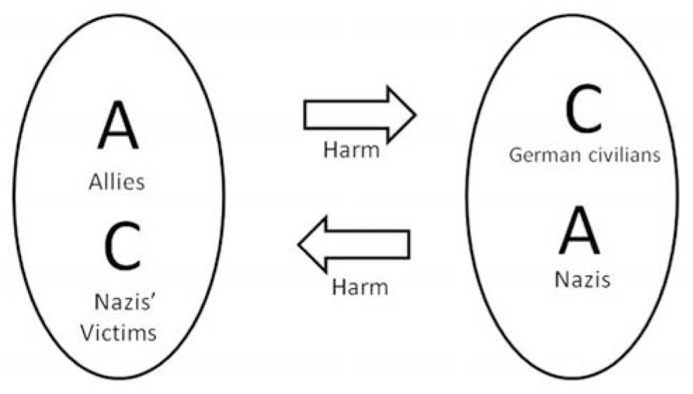
**Representation of difficult moral judgment.** In many difficult moral judgments, such as the bombing of civilians in Dresden in World War II, or the use of harsh methods of interrogation against terrorists, the victims are associated with the perpetrators and the perpetrators are associated with the victims. This induces a strong competition between perpetrator–victim category nodes making it difficult to reach a final judgment.

Serious moral dilemmas can also be explained by a connectionist model. These dilemmas require relatively high levels of cognitive processing because the victim is associated with a perpetrator and the perpetrator is associated with a victim. Thus each side sort of blocks a clear cut moral judgment (see **Figure 4**). The process involves a dynamic competition between victim–perpetrator representations (“they are victims but at the same time perpetrators”) which continuously compete. The bombing of civilians in Dresden in World War II, using harsh interrogation methods against terrorists, and policemen bullying cruel criminals, are some of the cases in which moral judgment is effortful and requires a great deal of processing.

For the system to settle into a stable state (e.g., reach a final moral judgment) the parallel and partially active Victim Vs. Perpetrator category nodes of each party must engage in a dynamic competition, with one gradually gaining activation and the other gradually dying off, as they suppress each other’s activation through inhibition.

Note that the model of a holistic dyad representation does not mean that the competing traits of the two parties (actor/patient) have to first of all be collapsed through a winner take all mechanism. Rather, it would seem more plausible that in serious moral dilemmas there are a multitude of representations also at the level of the dyad, and that the competition takes place at that level as well. This is naturally accommodated by a connectionist approach. “Dyad units” represent this domain on various levels of abstraction. The advantage of this perspective is that it accommodates complex situations better (e.g., when both sides are both right and wrong in certain respects).

## THE DYADIC PRINCIPLES AND THE FIRST YEAR OF LIFE

How can we judge so easily and effortlessly in so many different dyads when every dyad is embedded in different contexts and circumstances?

According to the thesis presented here, infants learn a set of procedures through the primary dyad that they later apply when facing real moral situations. What activates, stimulates, and makes possible, the learning of the complex mechanism involved in understanding the rules of the dyad?

Here we return to the theories of Bowlby and ethics of care with regard to the importance of the mother–infant relationship.

I propose that we know so much about dyads because we were once part of a dyad. Our internal schemas for children, adults, and the relation between them (→), are rooted in our early experiences. Moral cognitions and feelings draw on and grow out of such dyadic experiences.

According to the attachment approach, the strict principles of the primary dyad instantiated within our minds, impose themselves on the way we perceive and judge conflicted situations.

### INNATENESS

Darwin (1874), theorized that the social instincts originated in “parental and filial affections” (p. 95). Lakoff and Johnson (1999) write: “Brains tend to optimize on the basis of what they already have, to add only what is necessary. Over the course of evolution, newer parts of the brain have built on, taken input from, and used older parts of the brain.” (p. 43). Is it really plausible to suggest that if the infant-caregiver system can be put to work in the service of a parent protecting his child, the brain would build a new system to duplicate what it could already do in other social relations?

The representations of dyads and the expectations of how child–adult dyads should function, might be part of what Carey (2011) calls innate core cognition. Carey claims that the human capacity for conceptual understanding begins with the observation that evolution provides developmental primitives that are much richer than the sensorimotor representations that many hypothesize are the substrate for all learning. Some of these developmental primitives are embedded in systems of core cognition. However, in the absence of experience and learning through interactions with the environment, this capacity remains dormant.

In order for the genetically determined process to develop according to its blueprint, there must be an environmental stimulus that’s rich enough to trigger it. In other words, similarly to language acquisition, the practical experience in the interactions between the infant and the caregiver does not determine how the mind will work, but rather triggers it and causes it to operate in a predetermined way. It’s somewhat like a car: when we turn the key, the car operates like a car – not a boat – simply because it’s built like a car. However, if we don’t turn the key, nothing happens. The interactions between infant and caregiver are required in order to incorporate moral principle acquisition into the system, but the interactions don’t determine the content, form, or nature, of the specific morality.

Now, let us return to the studies showing that from 3 months of age, infants significantly preferred helpers over hinderers, suggesting the tendency to evaluate others by their third party prosocial and antisocial acts. These findings have been interpreted as reflecting unlearned innate abilities to discern and show appreciation of intention and acts in relation to another. In contrast, the attachment model of moral judgment explains these same findings as emerging out of the infant’s ability to *learn* a relation between dependent (weak, needy, seeking help) and independent (has resources to help/hinder). This ability may be innate (in a way similar to language competence) but must be triggered by the infant’s own experience. The infant’s total dependency, his constant biological and psychological needs, and seeking of help on the one hand, and the response or failure to respond to his needs on the other, is the context in which this learning takes place. Caregiver and infant reciprocally generate the chains and sequences pertaining to their mutual behavior, decipher, identify, and understand each other’s intentions, and the observing and experiencing infant learns this sequence, internalizes it, and is able to apply it as a criterion in future contexts. So when a puppet (red circle) attempts to climb the hill twice, each time falling back to the bottom, and on the third trial the climber is either bumped up the hill by a helper or bumped down the hill by a hinderer, the infant sees the target (red circle) as a “like-me” object who is in need of attaining a goal (climbing the hill). Based on his previous learning with her caregiver and surroundings the infant expects that animated objects will actively meet his needs and offer help. The helper meets the infant’s expectation, an expectation formed and forged by thousands of positive exchanges between the infant and the caregiver. An intention to hinder the climber and interfere with the achievement of the goal violates the infant’s expectations or reenacts experiences of disappointment thereby leading the infant to “stay away” from such objects. When infants see others needing to attain a goal they project that others have the same mental experience that goes with those behavioral states in the self and they expect the environment to help, not to hinder.

### UNIVERSALITY AND DIVERSITY IN MORAL JUDGMENT

The model has a powerful explanatory power for understanding the universal component of morality as opposed to the diversity cultural component.

It is possible that morality, like language, is grounded in two separate systems: knowledge and performance. Universal moral knowledge is based on the pre-verbal dyadic laws. This knowledge does not refer to any concrete dyad. It is “in our heads” before it is applied to the external world. It is based on our internal understanding of the dyad and its organizing principles. The second system is a performance system that is designed to apply this knowledge in the world. The performance system depends on the specific environment of the child.

Thus, the principles of the dyad represent the infant’s “starting point.” It could be thought of as the facet of the human mind that is responsible for diverse moral judgments having certain universal features in common. However, the fact that moral judgment follows a particular dyadic rule tells us little about the specific content of the agent’s judgment. Those issues relate to the performance system, the way in which we apply and practice the rules of the dyadic relations.

The “performance” component of our moral psychology is the functional characterization of the use of the dyadic principles in the course of construing moral situations out of social data and producing a moral judgment. Our moral performance cannot be determined independently of the social relational contexts in which it takes place. Some behaviors that people perceive as evil, actually have a moral basis in the psychology of the people who perform those acts (Rai and Fiske, 2011). For example, the cultural context determines which groups or people are privileged to be considered as part of the moral community. Only they will be construed as dependents in a case of harm. Only their suffering will activate the mechanisms of concern and empathy.

How can moral knowledge based on dyadic principles be claimed to be universal when moral systems and values differ so much from each other?

The dyadic principles, it has to be remembered, are not intended to be that which is common to all moral systems. These are principles that are intended to serve as a “toolkit” that a child acquires in order to learn how right/wrong judgments of all kinds are reached. It is the platform on which moral judgments are carried out, thought about, and understood. The dyadic structure itself tells us little about the content of the moral judgment. But it contains all the principles necessary for most moral judgments in different cultures and among a variety of agents. The differences among agents are encoded as assigning different weight to the various components of the dyad. Deciding according to which rule one decision will be considered morally superior to another in a given dyad is a complex matter. For example, social psychologists found again and again that empathy toward others probably increases if the “other” is similar to oneself in terms of ethnicity, gender, age, or cultural background (Wang et al., 2003). This means that a participant in a dyadic moral situation can be judged as dependent or child-like only if he is perceived as like-me in one way or another. Judging the victim as like-me might influence dyadic construal by, for example, exciting all nodes consistent with blame judgments toward the perceived perpetrator, and inhibiting all nodes inconsistent with them. Different strategies could be used. For instance, a “victim like-me” perception can inhibit excitation of child-like features of the perpetrator and excite the perpetrator’s adult-like features. The like-me criterion is decisive. However, this criterion is entirely subjective. It is part of the performance component of moral judgment.

## TESTING THE ATTACHMENT MODEL OF MORAL JUDGMENT

Beyond the model’s ability to explain a wide range of phenomena, it also gives rise to a number of new and distinctive predictions which future work could directly examine. Obviously, no experiment can illuminate the whole mental apparatus of our ability to make right/wrong judgments. Each question has to be broken down into empirically workable chunks.

Below are a few examples of important predictions derived from the model.

The model predicts that any given change in one node of the dyad component (A, C and →) will lead to changes in all other nodes, as the system works over time to maximally satisfy all of its constraints in parallel.

This might lead to several interesting predictions:

a) When, within a dyad, there are conflicting considerations (for example, harm not intended but identified with; harm mild but intended; baby face and intended harm; harm intended to one but meant to save several other victims) there is considerable tension between the nodes. This might reduce the system’s efficiency and slow down the process because it will take the system more time to reach a full gestalt of the dyad.
b) The model predicts that emotions and subjective preference will greatly influence the dyadic Gestalt and in specific ways. Stereotypes and prejudices against certain parties might block attributions of child-like qualities, exaggerate the perceived intention of wrongdoers, and reduce the perceived damage to victims.
c) The model predicts that a deep moral component based on the dyadic gestalt will be uniformly found in the structure of all moral judgments. For example, if, in a traditional culture, honor killing is permitted and encouraged, then the child-like quality of the victims will be less salient than in western culture. The same will be true for the adult-like quality of the perpetrator.

## CONCLUSION

I have described a rather basic hypothetical model for what is surely a rich and complex phenomenon. I suggest that the attachment approach to moral judgment has several features that make it especially attractive as a guide for research in moral psychology.

First, it holds that moral judgments are underpinned by internally represented principles or rules. Like sentimentalist accounts, the current model accepts that emotions, intuitions and unconscious processes, are central to the making of moral judgment. The added value of the attachment approach is that it specifies in detail what exactly goes on “in our head” when we follow our moral intuitions and how the entire procedure relates to cognitive processes. The intuitions and emotions behind moral judgments are rule based. By rules, I mean inferential devices for categorization, estimation, paired comparisons, and other judgmental tasks that go beyond the information given. The rule concept denotes an if–then relation of the type *if (cues) then (judgment)* (Kruglanski and Gigerenzer, 2011). One of the rules we use in judging moral situations is: if a party is perceived to be child-like (*cues*) then we judge that party as less accountable, less reprehensible, less responsible, less wrathful and so on.

Second, the important thing is that the rules apply to whole relations, not to specific harmful acts. We don’t judge an act as wrongdoing; we judge an entire relationship, a dyad. Wrongdoings are violations of our expectations of independents. Acts are judged as transgressions when an observer evaluates or senses that a dyad went wrong, violated an expected contingency.

Given the limited research base, this model – although reflecting available research evidence – serves primarily a heuristic function. I hope, nonetheless, that the model will inspire researchers to gain more empirical data on the mechanisms through which early attachment relations modulate moral judgments.

A deeper integration between moral knowledge, moral practice, emotions and cognitions, will require more explicit modeling and more empirical data, including neurobiology data on the mechanisms through which early attachment relations modulate moral judgments.

## Conflict of Interest Statement

The author declares that the research was conducted in the absence of any commercial or financial relationships that could be construed as a potential conflict of interest.

## Footnotes

1 For example it may be that the man’s mother would not condemn her son in the way that an impartial observer would because her love for her son makes it impossible for her to construe him as A (perpetrator).
